# Anti-Inflammatory Effect of Pineapple Rhizome Bromelain through Downregulation of the NF-κB- and MAPKs-Signaling Pathways in Lipopolysaccharide (LPS)-Stimulated RAW264.7 Cells

**DOI:** 10.3390/cimb43010008

**Published:** 2021-05-07

**Authors:** Orapin Insuan, Phornphimon Janchai, Benchaluk Thongchuai, Rujirek Chaiwongsa, Supaporn Khamchun, Somphot Saoin, Wimonrut Insuan, Peraphan Pothacharoen, Waraporn Apiwatanapiwat, Antika Boondaeng, Pilanee Vaithanomsat

**Affiliations:** 1 Department of Medical Technology, School of Allied Health Sciences, University of Phayao, Phayao 56000, Thailand; orapin.th@up.ac.th (O.I.); benchaluk.th@up.ac.th (B.T.); supaporn.kh@up.ac.th (S.K.); somphot.sa@up.ac.th (S.S.); 2 Unit of Excellence in Integrative Molecular Biomedicine, School of Allied Health Sciences, University of Phayao, Phayao 56000, Thailand; 3 Nanotechnology and Biotechnology Research Division, Kasetsart Agricultural and Agro-Industrial Product Improvement Institute (KAPI), Kasetsart University, Bangkok 10900, Thailand; aappmj@ku.ac.th (P.J.); aapwp@ku.ac.th (W.A.); aapakb@ku.ac.th (A.B.); 4 Department of Medical Technology, Faculty of Associated Medical Sciences, Chiang Mai University, Chiang Mai 50200, Thailand; rujirek.c@cmu.ac.th; 5 Department of Veterinary Technology, Faculty of Veterinary Technology, Kasetsart University, Bangkok 10900, Thailand; cvtwri@ku.ac.th; 6 Department of Biochemistry, Faculty of Medicine, Chiang Mai University, Chiang Mai 50200, Thailand; peraphan.p@cmu.ac.th; 7 Thailand Excellence Center for Tissue Engineering and Stem Cells, Department of Biochemistry, Faculty of Medicine, Chiang Mai University, Chiang Mai 50200, Thailand; 8 Center for Advanced Studies in Tropical Natural Resources, National Research University-Kasetsart University, Kasetsart University, Bangkok 10900, Thailand

**Keywords:** bromelain, inflammation, pro-inflammatory cytokines, NF-κB, MAPKs

## Abstract

Bromelain is a mixture of proteolytic enzymes derived from pineapple (*Ananas comosus*) fruit and stem possessing several beneficial properties, particularly anti-inflammatory activity. However, the molecular mechanisms underlying the anti-inflammatory effects of bromelain are unclear. This study investigated the anti-inflammatory effects and inhibitory molecular mechanisms of crude and purified rhizome bromelains on lipopolysaccharide (LPS)-induced inflammation in RAW 264.7 macrophage cells. RAW264.7 cells were pre-treated with various concentrations of crude bromelain (CB) or purified bromelain (PB), and then treated with LPS. The production levels of pro-inflammatory cytokines and mediators, including nitric oxide (NO), interleukin (IL)-6, and tumor necrosis factor (TNF)-α were determined by Griess and ELISA assays. The expressions of inducible nitric oxide synthetase (iNOS), cyclooxygenase (COX)-2, nuclear factor kappa B (NF-κB), and mitogen-activated protein kinases (MAPKs)-signaling pathway-related proteins were examined by western blot analysis. The pre-treatment of bromelain dose-dependently reduced LPS-induced pro-inflammatory cytokines and mediators, which correlated with downregulation of iNOS and COX-2 expressions. The inhibitory potency of PB was stronger than that of CB. PB also suppressed phosphorylated NF-κB (p65), nuclear factor of kappa light polypeptide gene enhancer in B-cells inhibitor alpha, extracellular signal-regulated kinases, c-Jun amino-terminal kinases, and p38 proteins in LPS-treated cells. PB then exhibited potent anti-inflammatory effects on LPS-induced inflammatory responses in RAW264.7 cells by inhibiting the NF-κB and MAPKs-signaling pathways.

## 1. Introduction

Bromelainis a mixture of proteolytic enzymes obtained from the fruit and stem of pineapple (*Ananas comosus*) [1,2] and is composed of different thiol endopeptidases and other components, such as phosphatases, glucosidases, peroxidase, cellulase, glycoprotein, and several protease inhibitors [3,4,[5,6]]. The biological properties of bromelain include antimicrobial, antithrombotic, anticancer, and anti-inflammatory effects [3,6]. Several studies have revealed that bromelain reduced the secretion of inflammatory cytokines, including interleukin (IL)-1β, IL-6, and tumor necrosis factor (TNF)-α in mouse macrophage cells [6,7]. Furthermore, bromelain potentially induces apoptosis in tumor cells by upregulation of p53 expression and initiation of the mitochondrial apoptotic pathway via increased Bax expression and cytochrome *c* release [8,9]. Additionally, bromelain inhibits nuclear factor-κB (NF-κB) translocation via G_2_/M arrest to apoptosis in human epidermoid carcinoma and melanoma cells [10]. Treatment of bromelain decreased the levels of cyclooxygenase-2 (COX-2) and vascular endothelial growth factor in hepatocellular carcinoma cells, resulting in reduced tumor neo-capillary density relative to that of untreated cells [11]. It has been reported that bromelain exhibited anti-inflammatory effects in lipopolysaccharide (LPS) on human U937 macrophages by suppressing pro-inflammatory cytokines, chemokines, and the cyclooxygenase pathway [12].

Inflammation is a complex process that has a pivotal role in the development of several diseases, including arthritis, cardiovascular diseases, diabetes mellitus, metabolic syndromes, and cancers [13,14]. In the inflammatory process, the overproduction of pro-inflammatory cytokines, and inflammatory mediators, such as TNF-α, IL-6, nitric oxide (NO), and prostaglandin E_2_ (PGE_2_) secreted from macrophage cells, are involved in the pathogenesis of inflammatory diseases [15,16,17]. Inducible nitric oxide synthase (iNOS) andCOX-2 are the key regulator enzymes associated with the production of NO and PGE_2_, respectively [18,19,20]. The expression of these pro-inflammatory cytokines is mostly regulated by two main pathways: The mitogen-activated protein kinases (MAPKs) and NF-κB-signaling pathways [21,22,23] and are also known as the myeloid differentiation factor 88 (MyD88)-dependent pathway, and MyD88-independent pathway [24,25], respectively. Commonly, these two pathways are stimulated by LPS in macrophage cells and are downstream of toll-like receptor (TLR) 4 signaling. NF-κB, a transcription factor composed of p65 and p50 subunits, regulates the transcription of multiple genes of pro-inflammatory mediators and cytokines [26,27]. In a normal status, NF-κB is bound with the inhibitory kappa B (IκB) to form the NF-κB–IκB complex in the cytoplasm. In the case of inflammatory responses, NF-κB is phosphorylated, translocated into the nucleus, and bound to the promoter regions of pro-inflammatory genes, including iNOS, COX-2, IL-1β, IL-6, and TNF-α [28,29,30]. The MAPKs pathway includes extracellular signal-regulated kinase 1/2 (ERK1/2), c-Jun N-terminal kinase (JNK), and p38 [31] and controls the expression of pro-inflammatory mediators and cytokines by sequential phosphorylation [32,33]. Therefore, inhibitors of the NF-κB and MAPKs pathways might be used as therapeutic agents for treatment of inflammatory diseases.

Normally, the fruit of pineapple is used as food, whereas the stem and rhizome are a waste product and inexpensive. The pineapple stem contains a high amount of bromelain. During pineapple processing, the crown, stem, and rhizome are cut off before peeling, large amounts of waste are not utilized. Pineapple wastes are observed to have potential as raw materials that can be converted into value-added products. Therefore, the possibility of utilizing their wastes is worthy of investigation. Additionally, there have been only few studies on the molecular inhibitory effect of bromelain through the NF-κB and MAPKs pathways. Therefore, the present study aimed to explore the anti-inflammatory effects and molecular mechanisms underlying the inhibitory effects of crude and purified rhizome bromelains on LPS-induced inflammation in RAW 264.7 macrophages.

## 2. Results

### 2.1. Characterization of Bromelain Extracts

**Figure 1:**
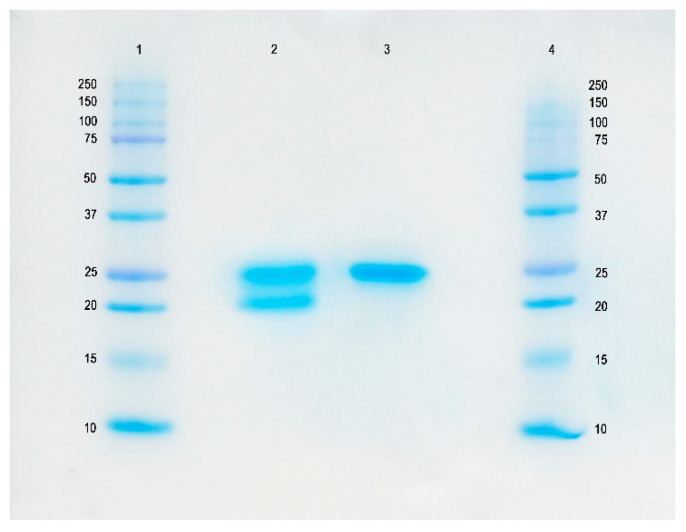
SDS-PAGE of the 0.1 mg protein containing crude and purified extracts: Lanes 1 and 4, protein markers; Lane 2, crude bromelain (CB); Lane 3, purified bromelain (PB).

The pineapple rhizome bromelain extracts were subjected to protein quantitative determination and protease activity assay. The results showed the protease activity of crude bromelain (CB) (2767.09 CDU/g) and purified bromelain (PB) (78,934.74 CDU/g), which clearly corresponded with the protein concentrations (0.09 g/g and 0.98 g/g, respectively). The molecular weights of CB and PB determined by sodium dodecyl sulfate-polyacrylamide gel electrophoresis (SDS–PAGE) were approximately 25 KDa (Figure 1). The band intensity of the single PB was more intense than that of the CB, which indicated that most of the protein impurities were removed in the purification step and thus gave a single band for the PB on SDS–PAGE.

### 2.2. Cytotoxic Effects of Bromelain Extracts on RAW264.7 Cells

**Figure 2:**
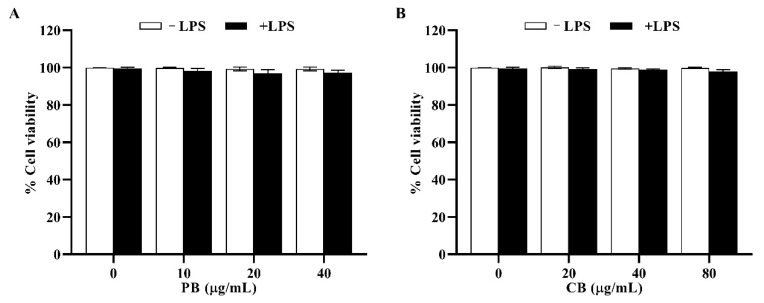
The effect of bromelain on the cell viability of RAW264.7 macrophage cells. The cells (2 × 10^4^ cells/well) were pre-treated with indicated concentrations of (**A**): purified bromelain (PB) and (**B**): crude bromelain (CB) for 2 h and stimulated with or without LPS for 22 h. Cell viability was performed by MTT assay. The results are expressed as the mean ± SD (*n* = 3).

The effects of bromelain extracts on RAW264.7 cell viability are shown in Figure 2. The results showed that both PB (10–40 µg/mL) and CB (20–80 µg/mL) with, or without, LPS (100 ng/mL) had no effect on the viability of RAW264.7 macrophage cells (% cell survival > 80%). On the other hand, treatment of LPS in a combination with PB or CB (100–200 µg/mL) exhibited the toxic effect on RAW264.7 cells (cell viability < 70%) and 10% DMSO treatment (toxic control) decreased the viability of RAW264.7 cells to 27.5% (data not shown). Therefore, these optimal concentrations of bromelain extracts were used in the subsequent experiment.

### 2.3. Effects of Bromelain Extracts on LPS-Induced NO Production and Expressions of iNOS and COX-2 in RAW264.7 Cells

**Figure 3:**
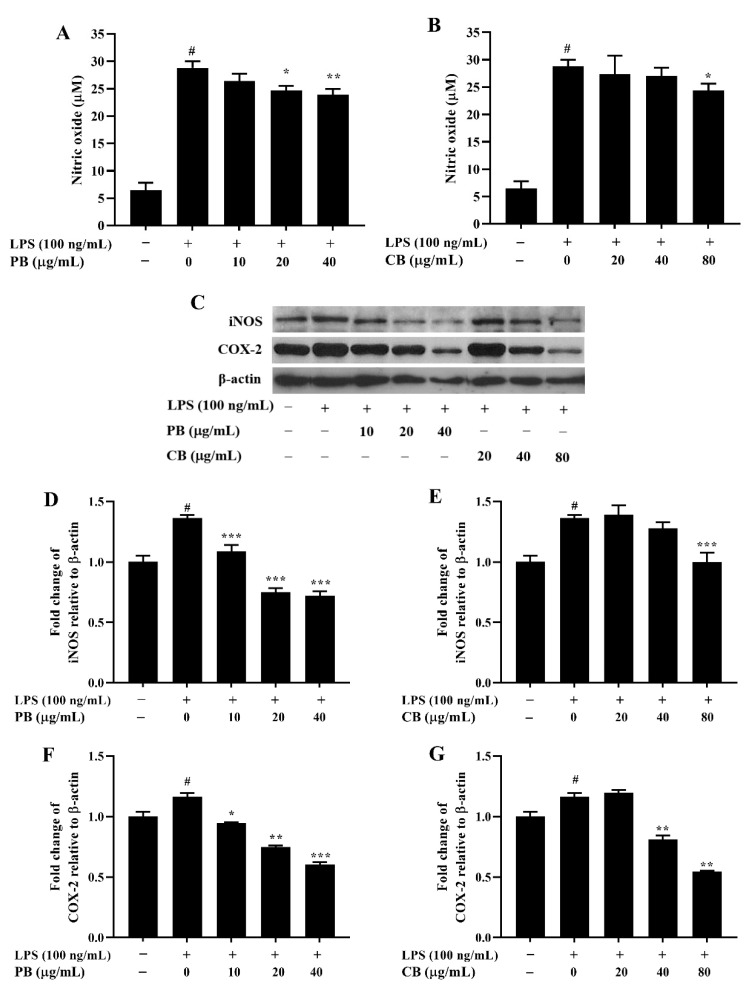
The effect of bromelain on the production of NO and the expressions of iNOS and COX-2 proteins in LPS-induced RAW264.7 macrophage cells. The cells were treated with various concentrations of bromelain for 2 h and stimulated with LPS (100 ng/mL) for 22 h. (**A**,**B**): The level of NO in cell culture supernatant was determined by Griess assay; (**C**–**G**): The expression levels of iNOS and COX-2 proteins were determined by western blot analysis, PB: purified bromelain, CB: crude bromelain. The results are expressed as the mean ± SD (*n* = 3). ^#^ *p* < 0.05 indicates a significant difference from the LPS-untreated cells, * *p* < 0.05, ** *p* < 0.005, and *** *p* < 0.001 indicate significant differences from the LPS alone.

To investigate the anti-inflammatory activity of bromelain extracts, we first measured the production of NO in LPS-stimulated RAW264.7 cells. As shown in Figure 3A, the NO level in the LPS-treated group showed a marked increase relative to that in the control group. The release of NO was inhibited by PB in a dose-dependent manner. However, the level of NO in CB was significantly decreased only at the concentration of 80 µg/mL relative to that in the LPS-treated group (Figure 3B). The production of NO is directly related to upregulation of iNOS expression. Therefore, the effect of bromelain extracts on LPS-induced iNOS protein expression was examined by performing western blot analysis. In a similar pattern, the iNOS protein level was significantly increased by LPS stimulation, and bromelain extracts suppressed the expression of iNOS protein in a dose-dependent manner (Figure 3C–E). Moreover, downregulation of COX-2 protein expression, a key regulation enzyme in the production of PGE_2_, was also observed after treatment with different concentrations of PB and CB in a dose-dependent manner (Figure 3F,G).

### 2.4. Effects of Bromelain Extracts on LPS-Induced IL-6 and TNF-α in RAW264.7 Cells

**Figure 4:**
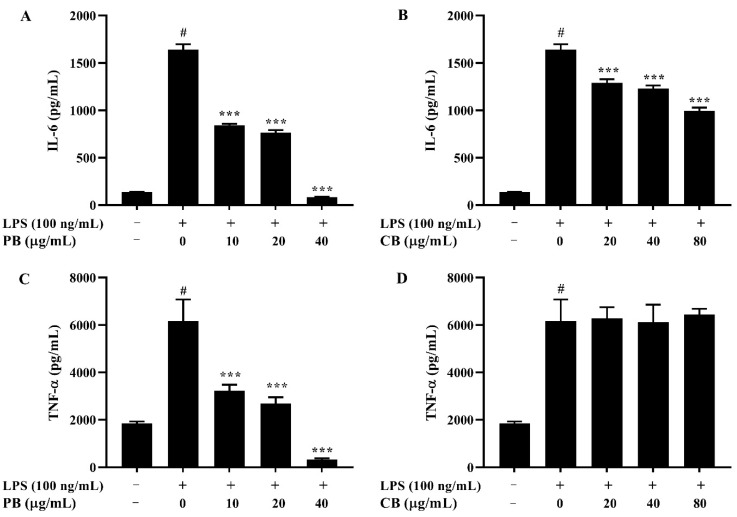
The effect of bromelain on the production of pro-inflammatory cytokines in LPS-induced RAW264.7 macrophage cells. The cells (2 × 10^4^ cells/well) were treated with various concentrations of bromelain for 2 h and stimulated with LPS (100 ng/mL) for 22 h. Cytokine levels of IL-6 and TNF-α in the culture supernatant were measured using an ELISA kit. (**A**,**C**): purified bromelain (PB); (**B**,**D**): crude bromelain (CB). The results are expressed as the mean ± SD (*n* = 3). ^#^ *p* < 0.05 indicates a significant difference from the LPS-untreated cells, *** *p* < 0.001 indicate significant differences from the LPS alone.

As the treatment of bromelain extracts inhibited expressions of the pro-inflammatory mediators NO and iNOS, the effects of bromelain extracts on the pro-inflammatory cytokines, including IL-6 and TNF-α, were examined by ELISA. The results showed that the levels of IL-6 and TNF-α were significantly higher in the LPS-treated group than in the control group (Figure 4). Pre-treatment with various doses of PB significantly decreased LPS-stimulated IL-6 and TNF-α production in a dose-dependent manner (Figure 4A,C). The produced level of IL-6 was dramatically reduced by CB relative to that in the LPS-treated group in a concentration-dependent manner (Figure 4B). However, the production level of TNF-α was not changed (Figure 4D).

### 2.5. Effects of Bromelain Extracts on LPS-Induced Expression of NF-κB Pathway-Related Proteins

**Figure 5:**
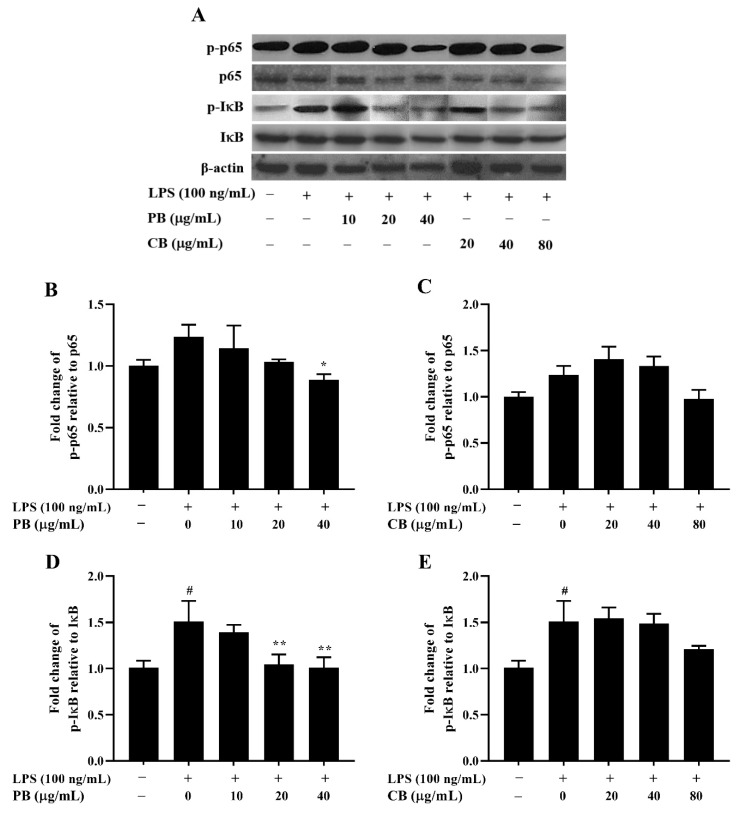
Inhibitory effect of bromelain on the expression of NF-κB pathway-related protein in LPS-induced RAW264.7 macrophage cells. The cells (2 × 10^6^ cells/well) were treated with various concentrations of bromelain for 2 h and stimulated with LPS (100 ng/mL). (**A**): The protein levels of phospho and non-phospho forms of the NF-κB signaling molecules, including p65 and IκB were determined in cell lysates using western blot analysis; (**B**–**E**): Phosphorylation band densities of p65 and IκB relative to the total form in RAW264.7 macrophage cells; PB: purified bromelain, CB: crude bromelain. The results are expressed as the mean ± SD (*n* = 3). ^#^ *p* < 0.05 indicates a significant difference from the LPS-untreated cells, * *p* < 0.05 and ** *p* < 0.005 indicate significant differences from the LPS alone.

To investigate the molecular mechanism underlying the inhibitory effect of bromelain on LPS-stimulated inflammation in RAW264.7 macrophage cells, the expression of NF-κB, an important transcription factor associated with inflammatory signaling transduction, was measured by using western blot analysis. As shown in Figure 5A, the total expression levels of the NF-κB pathway-related proteins, NF-κB (p65 subunits) and inhibitor of κB (IκB)-α, in the LPS-treated group were not changed relative to those in the control group. Interestingly, the phosphorylation of p65 and IκB-α were markedly inhibited by treatment with various concentrations of PB (10–40 µg/mL) in a dose-dependent manner (Figure 5B,D). On the other hand, CB did not inhibit LPS-induced phosphorylation of p65 and IκB-α (Figure 5C,E)

### 2.6. Effects of Bromelain Extracts on LPS-Induced Phosphorylation of the MAPKs Pathway

**Figure 6:**
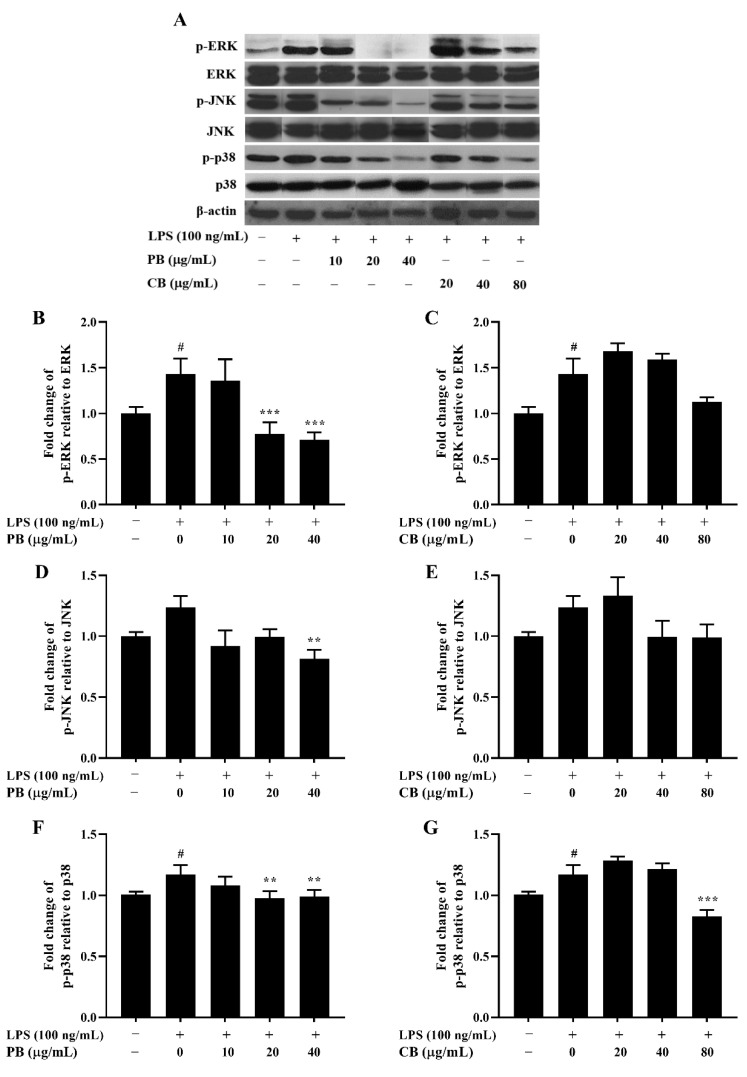
Inhibitory effect of bromelain on MAPK phosphorylation in LPS-induced RAW264.7 macrophage cells. The cells (2 × 10^6^ cells/well) were treated with various concentrations of bromelain for 2 h and stimulated with LPS (100 ng/mL). (**A**): The protein levels of phospho and non-phospho forms of the MAPK signaling molecules, including ERK, JNK, and p38 were determined in cell lysates using western blot analysis; (**B**–**G**): Phosphorylation band densities of ERK, JNK, and p38 relative to the total form in RAW264.7 macrophage cells; PB: purified bromelain, CB: crude bromelain. The results are expressed as the mean ± SD (*n* = 3). ^#^ *p* < 0.05 indicates a significant difference from the LPS-untreated cells, ** *p* < 0.005 and *** *p* < 0.001 indicate significant differences from the LPS alone.

MAPKs-signaling is involved in LPS-stimulated iNOS and COX-2 expression in activated macrophages and has a critical role in the activation of NF-κB [30]. The MAPKs pathway, including ERK1/2, JNK, and p38, were investigated in this study. As presented in Figure 6, the total expression levels of ERK1/2, JNK, and p38 were unchanged by CB and PB relative to those in the LPS-treated group. The phosphorylation of ERK1/2, JNK, and p38 were increased by LPS stimulation. Treatment of purified bromelain dose-dependently attenuated LPS-induced phosphorylation of ERK1/2, JNK, and p38. In contrast, CB had no effect on the phosphorylation of ERK1/2 and JNK, while phosphorylation of p38 was suppressed in LPS-induced inflammation.

## 3. Discussion

The inhibition of inflammatory mediators and cytokines is considered to be an effective therapeutic strategy for the treatment of inflammatory diseases. Recently, bromelain was shown to be a major constituent derived from pineapple (*A. comosus*) [1,2] and potentially has biological properties, particularly anti-inflammatory activity [6]. However, the inhibitory mechanism of bromelain on LPS-induced inflammation is unclear. In the present study, we examined the inflammatory effects of CB and PB extracted from pineapple rhizomes, and the molecular mechanisms underlying the anti-inflammatory effect of bromelain on LPS-stimulated RAW264.7 cells were determined. Macrophages are innate immune cells that have a vital role in the inflammatory process [34,35]. They promote immune responses by increasing the production of inflammatory mediators and pro-inflammatory cytokines, including TNF-α, IL-6, IL-1β, NO, and PGE_2_ [15,16,17]. For anti-inflammatory agent screening, the model of LPS-induced inflammatory responses in RAW264.7 cells has been commonly used [36].

The excessive expression of pro-inflammatory cytokines from macrophages is directly linked to the pathogenesis of inflammatory diseases [16,17,37]. IL-6, a potential pro-inflammatory cytokine, triggers numerous important physiological functions, particularly acute and chronic inflammation [38]. TNF-α has a major role in regulating immune responses and inflammation through activation of TNF receptors and related pathways, such as NF-κB and MAPKs [39]. The activation of NF-κB leads to phosphorylation and translocation of NF-κB into the nucleus and binding to the promoter regions of pro-inflammatory genes, including iNOS, COX-2, IL-1β, IL-6, and TNF-α [40,41,42]. Upregulated expressions of iNOS and COX-2 genes result in increased production of NO, and PGE_2_, respectively [43]. Bromelain has been shown to cause decreased secretion of IL-1β, IL-6, and TNF-α under the condition of inflammation-induced overproduction of cytokines [6,7]. Additionally, bromelain downregulated the expression of COX-2 and PGE_2_ levels in murine microglial cells and human monocytic leukemia cell lines [44]. In the present study, we showed that LPS significantly increased production of NO, IL-6, and TNF-α in RAW264.7 macrophage cells. PB that contains high protease activity significantly suppressed the levels of LPS-induced cytokines and production of mediators, including NO, IL-6, and TNF-α, in a dose-dependent manner, which correlated with downregulation of iNOS and COX-2 expressions. However, the effects of CB on the reduction level of pro-inflammatory cytokines and mediators were observed to be less than those of PB. The levels of NO and IL-6, but not TNF-α were significantly decreased when treated with CB. The composition of bromelain was based on the source and purification method. Compared with fruit-derived bromelain, stem-derived bromelain contains a higher amount of protease [6]. The proteolytic action has been reported to be responsible for numerous pharmacological activities [3,45]. A previous study has shown that bromelain, a mixture of cysteine proteases from pineapple stems, inhibited ERK-2 activation in T cells and the inhibitory activity of bromelain was dependent on its proteolytic activity [46]. Additionally, the beneficial effects of bromelain may not be due to a single proteolytic fraction, and is probably due to multiple factors [45]. Our results indicated that bromelain has a protective effect on LPS-induced inflammation in RAW264.7 cells. The anti-inflammatory activity of PB was more potent than that of CB, and the inhibitory effect of bromelain on NO production depended on reduction of iNOS expression.

Although the anti-inflammatory effects of bromelain have been reported, the underlying mechanisms remain unclear. Therefore, the anti-inflammatory effect of bromelain on related pathways in LPS-induced RAW264.7 cells were investigated in this study. Pro-inflammatory cytokines, such as IL-1β, IL-6, and TNF-α are directly associated with the intracellular-signaling pathways, NF-κB and MAPKs [47,48]. The transcription factor, NF-κB, is a heterodimer consisting of p50/p65. Stimulation of NF-κB in macrophages results in degradation of IκB and dissociation of NF-κB from its inhibitory complex. The free form of NF-κB is then translocated into the nucleus and promotes transcription of the corresponding pro-inflammatory genes, including COX-2, iNOS, IL-1β, IL-6, and TNF-α [27,49,50]. Our results revealed that only PB reduced phosphorylation of NF-κB p65 and IκB-α in LPS-induced RAW264.7 cells. We suggest that PB could decrease the levels of pro-inflammatory cytokines and mediators through inhibition of NF-κB activation. MAPKs are serine/threonine protein kinases, composed of three major sub-families, including ERK1/2, JNK, and p38. LPS activated the MAPK-signaling pathway in macrophages by binding to TLR4 [51]. Several studies have demonstrated that activation of MAPKs by LPS is involved in increasing the level of inflammatory mediators through stimulation of NF-κB [52]. The present study demonstrated that PB significantly decreased expression of phosphorylated ERK, JNK, and p38 in LPS-activated RAW264.7 cells, which indicated that bromelain-mediated inhibition of inflammatory responses might be accomplished by downregulating expression of MAPK pathway-related proteins. Taken together, PB showed strong anti-inflammatory effects against LPS-induced inflammation in RAW264.7 cells via the NF-κB and MAPKs-signaling pathways.

## 4. Materials and Methods

### 4.1. Chemicals and Reagents

Lipopolysaccharide (LPS) from *Escherichia coli* serotype (O111:B4) and 3-(4,5-dimethylthiazol-2-yl)-2,5-diphenyltetrazolium bromide (MTT) were purchased from Sigma-Aldrich (St. Louis, MO, USA). Dulbecco’s modified Eagle’s medium (DMEM), fetal bovine serum, streptomycin, and penicillin were obtained from Invitrogen Gibco (Grand Island, NY, USA). Griess reagent was purchased from Invitrogen, Thermo Fisher Scientific, Inc. (Eugene, Oregon, USA). TNF-α and IL-6 enzyme-linked immunosorbent assay (ELISA) kits were purchased from R&D Systems, Inc. (Minneapolis, MN, USA). Purchases of iNOS, COX-2, p-NF-κB p65, NF-κB p65, pI-κB, I-κB, p-ERK, ERK, p-JNK, JNK, p-p38, p38, and β-actin antibodies were made from Cell Signaling Technology (Beverly, MA, USA) and Thermo Fisher Scientific, Inc. (Rockford, IL, USA).

### 4.2. Preparation of Bromelain Extracts from Pineapple Rhizome

Pineapple (*A. comosus*) rhizome, used as a raw material for bromelain preparation, was kindly provided from the canned pineapple manufacturer in Chonburi Province, Thailand. Pineapple rhizome was chopped into small pieces and blended with water (1:1 *w*/*v*) to obtain a pineapple slurry. The crude and purified bromelain were prepared following protocols 1, and 2, respectively, shown below.

Protocol 1: the finely powdered ammonium sulfate was added gradually into a pineapple slurry to obtain 50% saturation with continuous stirring for 1 hand aging at 4 °C overnight. The crude bromelain (CB) precipitate was recovered by centrifugation at 10,000× g for 30 min at 4 °C and dried.

Protocol 2: the finely powdered ammonium sulfate was added gradually into a pineapple slurry to obtain 40–80% saturation with continuous stirring for 1 hand aging at 4 °C overnight. The purified bromelain (PB) precipitate was recovered by centrifugation at 10,000× g for 30 min at 4 °C and dried. Finally, 7.6 g of dry protein was obtained. The protein content of the separated fraction reached 99%, the purity by electrophoresis of the obtained fraction was 100%, and the activity recovery was 95.5%.

### 4.3. Characterization of Bromelain Extracts

Protein content determination in bromelain extracts was performed by using the Lowry method [53]. The protein content was calculated by using bovine serum albumin as a standard. Protease activity in the bromelain extracts was quantitatively determined according to Ketnawa et al. (2009) by using the casein digestion unit (CDU) method with tyrosine as a standard [54]. The reaction mixture contained 5.0 mL 0.6% casein (*w*/*v*) and 1 mL enzyme appropriately diluted in 0.3 M l-cysteine and 0.006 M EDTA. The mixture was incubated at 37 °C for 10 min before starting the reaction by adding casein solution. The reaction was stopped by adding 5.0 mL of trichloroacetic acid. Precipitated protein was removed by centrifugation at 7100× g for 20 min. The absorbance of the clear supernatant was measured at 275 nm. One unit of protease activity was defined as the amount of enzyme-releasing product equivalent to 1 μg of tyrosine min^−1^ mL^−1^ under the assay conditions.

The molecular weight of extracted bromelain was determined by SDS–PAGE according to the method of Laemmli (1970) [55]. The sample was mixed at a ratio of 1:1 with the sample buffer (0.5 M Tris-HCl, pH 6.8, containing 4% SDS and 20% glycerol). For the reduction condition, 10% of β-mercaptoethanol was added to the sample buffer. The samples (20 µg of protein) were loaded into the polyacrylamide gel (10% running and 4% stacking gels) and subjected to electrophoresis at a constant current of 15 mA/gel. After separation, the protein was stained with staining solution (0.02% Coomassie Brilliant Blue R-250) and de-stained with an acetic-acid–methanol solution.

### 4.4. Cell Culture

RAW264.7 mouse macrophage cell line was obtained from American Type Culture Collection (ATCC^®^ number TIB-71) and cultured in DMEM supplemented with 10% fetal bovine serum and 100 units/mL penicillin–streptomycin under a humidified (95%) atmosphere of 5% CO_2_ at 37 °C. Cells from passages 3–7 were used in the experiments. The cells were pre-treated with various concentrations of PB (10, 20, and 40 µg/mL) or CB (20, 40, and 80 µg/mL) for 2 h and then treated with 100 ng/mL LPS. After 24 h treatment, the cell viability and expressions of protein were determined. The levels of NO, IL-6, and TNF-α in culture supernatants were determined.

### 4.5. Cell Viability Assay

The cytotoxicity of bromelain extracts to RAW264.7 cells was measured by performing the MTT assay. RAW264.7 macrophage cells were seeded into a 96-well plate at a density of 2 × 10^4^ cells/well (cell viability > 95%). After 24 h, the cells were pre-treated with various concentrations of PB or CB for 2 h and subsequently treated with100 ng/mL LPS for 22 h. Next, the cells were incubated with MTT solution at the final concentration of 0.5 mg/mL for 4 h at 37 °C. Then, the supernatant was removed, 100 µL of dimethyl sulfoxide was added to dissolve the formazan crystals, and absorbance at 540 nm was determined by using a microplate reader. Untreated cells with a cell viability of 100% were used as the control. The cell viability of the treated wells was calculated relative to that of the control as a percentage.

### 4.6. Measurement of Nitric oxide and Cytokines Productions

RAW264.7 macrophage cells (2 × 10^4^ cells/well) were seeded into a 96-well plate and treated with various concentrations of bromelain extracts. After 2 h of treatment, the cells were incubated with LPS (100 ng/mL) for 22 h. The level of NO production in the cell culture supernatant was measured by using a Griess reagent kit. Briefly, Griess reagent (0.1% *N*-(1-naphthyl) ethylenediamine dihydrochloride/1% sulfanilic acid in 5% phosphoric acid) was mixed with cell culture medium and incubated at room temperature in the dark for 30 min. The concentration of NO was determined by using a microplate reader to measure the absorbance at 542 nm. For the measurement of pro-inflammatory cytokines, the cells were seeded into a 6-well plate (2 × 10^6^ cells/mL) and incubated in the presence of different concentrations of bromelain extracts for 2 h, followed by stimulation with LPS for 22 h. The levels of IL-6 and TNF-α were detected by using an ELISA assay kit (R&D Systems) following the manufacturer’s instructions. The absorbance at a wavelength of 450 nm was determined by using a microplate reader.

### 4.7. Western Blot Analysis

RAW264.7 macrophage cells were seeded into 6-well plates at a concentration of 2 × 10^6^ cells/mL. After 24 h, the cells were treated with different concentrations of bromelain extracts for 2 h and subsequently treated with LPS (100 ng/mL) for 22 h. The cells were harvested and washed with cold phosphate buffer saline. Total proteins were extracted from cells using Laemmli lysis buffer (Bio-Rad Laboratories, Inc., CA, USA), and the protein concentration was quantified by using a Bradford assay kit (Thermo Fisher Scientific, Inc., IL, USA). Western blot analysis was performed according to a standard protocol. Briefly, 30 µg of proteins was separated by 10% SDS–PAGE and transferred to nitrocellulose membranes. The membranes were blocked in 5% non-fat dry milk in TRIS-buffered saline (TBS) and consequently incubated with primary rabbit monoclonal antibodies against iNOS, COX-2, p-NF-κB p65, NF-κB p65, pI-κB, I-κB, p-ERK, ERK, p-JNK, JNK, p-p38, p38, and β-actin, which served as a loading control at 4 °C overnight. The membranes were then washed three times with 0.05% Tween 20-TBS and incubated with horseradish peroxidase-conjugated secondary antibodies at room temperature for 1 h. Protein bands were detected by using an enhanced chemiluminescence reagent (Bio-Rad Laboratories, Inc., CA, USA) and then exposed to X-ray film. Band intensity data was obtained by using ImageJ software (https://imagej.nih.gov/ij, accessed on 1 September 2020).

### 4.8. Statistical Analysis

The results were expressed as the mean ±SD from triplicate samples of three independent experiments. Differences between conditions were evaluated by one-way ANOVA analysis. Statistical significance was accepted for *p* values < 0.05.

## 5. Conclusions

Here, we demonstrated that rhizome bromelain extract, including CB and PB exert the inhibitory effects against LPS-stimulated inflammatory responses in RAW264.7 macrophage cells. PB exhibited more potent anti-inflammatory effects than CB. Inhibition of iNOS and COX-2 expression by PB reduced NO, IL-6, and TNF-α production. The anti-inflammatory effect of bromelain on LPS-induced inflammatory responses was associated with decreased expression of NF-κB and MAPKs-signaling pathway-related proteins. These results indicated that PB could be a potential therapeutic agent against inflammatory diseases.

## Data Availability

Data is contained within the article.
