# Correction: Insuan et al. Anti-Inflammatory Effect of Pineapple Rhizome Bromelain through Downregulation of the NF-κB- and MAPKs-Signaling Pathways in Lipopolysaccharide (LPS)-Stimulated RAW264.7 Cells. *Curr. Issues Mol. Biol*. 2021, *43*, 93–106

**DOI:** 10.3390/cimb48010017

**Published:** 2025-12-24

**Authors:** Orapin Insuan, Phornphimon Janchai, Benchaluk Thongchuai, Rujirek Chaiwongsa, Supaporn Khamchun, Somphot Saoin, Wimonrut Insuan, Peraphan Pothacharoen, Waraporn Apiwatanapiwat, Antika Boondaeng, Pilanee Vaithanomsat

**Affiliations:** 1 Department of Medical Technology, School of Allied Health Sciences, University of Phayao, Phayao 56000, Thailand; orapin.th@up.ac.th (O.I.); benchaluk.th@up.ac.th (B.T.); supaporn.kh@up.ac.th (S.K.); somphot.sa@up.ac.th (S.S.); 2 Unit of Excellence in Integrative Molecular Biomedicine, School of Allied Health Sciences, University of Phayao, Phayao 56000, Thailand; 3 Nanotechnology and Biotechnology Research Division, Kasetsart Agricultural and Agro-Industrial Product Improvement Institute (KAPI), Kasetsart University, Bangkok 10900, Thailand; aappmj@ku.ac.th (P.J.); aapwp@ku.ac.th (W.A.); aapakb@ku.ac.th (A.B.); 4 Department of Medical Technology, Faculty of Associated Medical Sciences, Chiang Mai University, Chiang Mai 50200, Thailand; rujirek.c@cmu.ac.th; 5 Department of Veterinary Technology, Faculty of Veterinary Technology, Kasetsart University, Bangkok 10900, Thailand; cvtwri@ku.ac.th; 6 Department of Biochemistry, Faculty of Medicine, Chiang Mai University, Chiang Mai 50200, Thailand; peraphan.p@cmu.ac.th; 7 Thailand Excellence Center for Tissue Engineering and Stem Cells, Department of Biochemistry, Faculty of Medicine, Chiang Mai University, Chiang Mai 50200, Thailand; 8 Center for Advanced Studies in Tropical Natural Resources, National Research University-Kasetsart University, Kasetsart University, Bangkok 10900, Thailand

## Figure Correction

In the original publication, the Western blot images presented in Figures 3C, 5A and 6A contained errors in image presentation [1]. The authors sincerely apologize for this inadvertent mistake and have requested that the figures be corrected by replacing the previously published figures with the appropriate versions. To ensure the reproducibility and reliability of the experimental results, the authors have carefully re-examined the data and repeated the experiments, with the aim of improving quality of the reported results. The corrected versions of Figure 3C, Figure 5A and Figure 6A are provided below. The authors state that the scientific conclusions are unaffected. This correction was approved by the Academic Editor. The original publication has also been updated. 

## Figures and Tables

**Figure 3:**
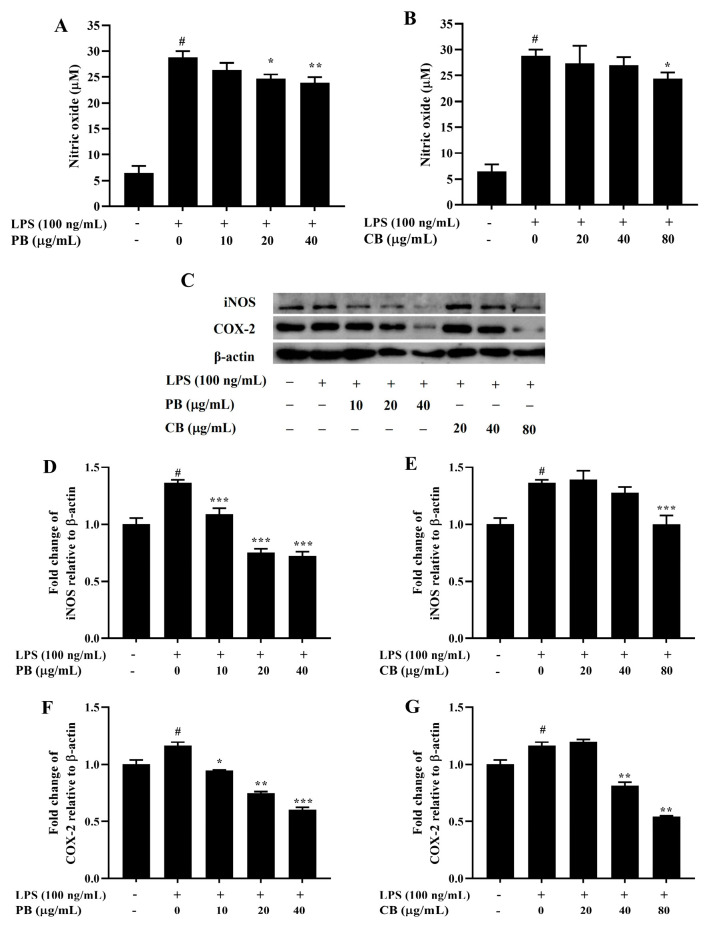
The effect of bromelain on the production of NO and the expressions of iNOS and COX-2 proteins in LPS-induced RAW264.7 macrophage cells. The cells were treated with various concentrations of bromelain for 2 h and stimulated with LPS (100 ng/mL) for 22 h. (**A**,**B**): The level of NO in cell culture supernatant was determined by Griess assay; (**C**–**G**): The expression levels of iNOS and COX-2 proteins were determined by western blot analysis, PB: purified bromelain, CB: crude bromelain. The results are expressed as the mean ± SD (*n* = 3). ^#^ *p* < 0.05 indicates a significant difference from the LPS-untreated cells, * *p* < 0.05, ** *p* < 0.005, and *** *p* < 0.001 indicate significant differences from the LPS alone.

**Figure 5:**
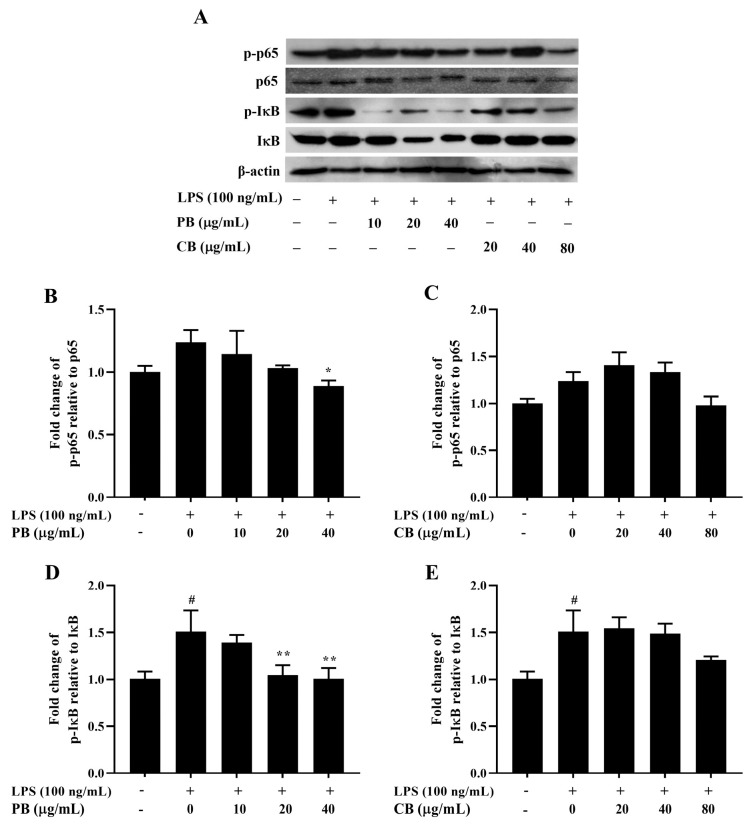
Inhibitory effect of bromelain on the expression of NF-κB pathway-related protein in LPS-induced RAW264.7 macrophage cells. The cells (2 × 10^6^ cells/well) were treated with various concentrations of bromelain for 2 h and stimulated with LPS (100 ng/mL). (**A**): The protein levels of phospho and non-phospho forms of the NF-κB signaling molecules, including p65 and IκB were determined in cell lysates using western blot analysis; (**B**–**E**): Phosphorylation band densities of p65 and IκB relative to the total form in RAW264.7 macrophage cells; PB: purified bromelain, CB: crude bromelain. The results are expressed as the mean ± SD (*n* = 3). ^#^ *p* < 0.05 indicates a significant difference from the LPS-untreated cells, * *p* < 0.05 and ** *p* < 0.005 indicate significant differences from the LPS alone.

**Figure 6:**
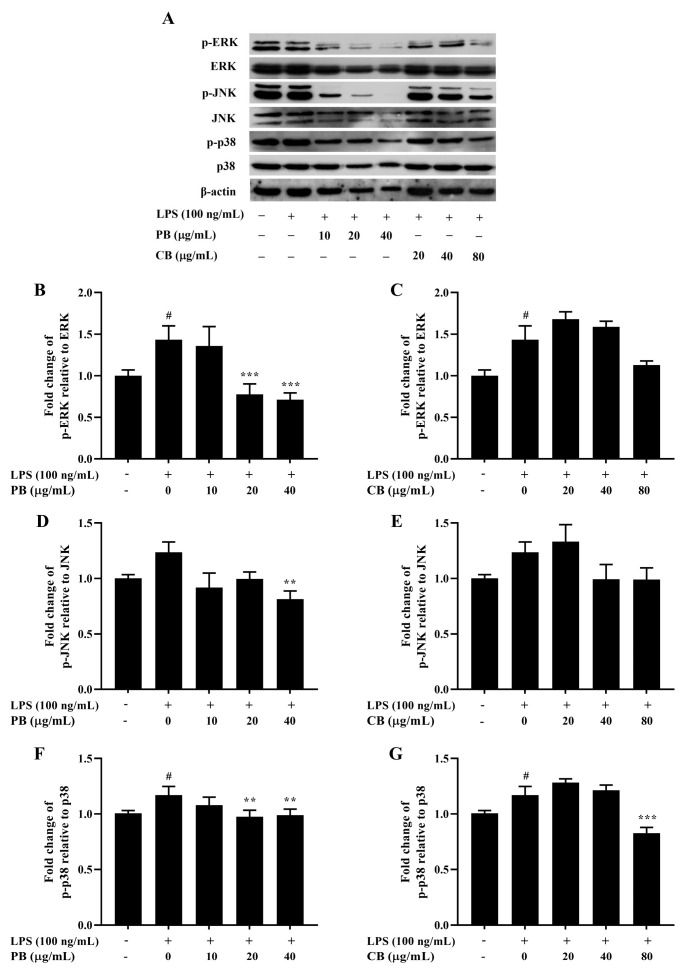
Inhibitory effect of bromelain on MAPK phosphorylation in LPS-induced RAW264.7 macrophage cells. The cells (2 × 10^6^ cells/well) were treated with various concentrations of bromelain for 2 h and stimulated with LPS (100 ng/mL). (**A**): The protein levels of phospho and non-phospho forms of the MAPK signaling molecules, including ERK, JNK, and p38 were determined in cell lysates using western blot analysis; (**B**–**G**): Phosphorylation band densities of ERK, JNK, and p38 relative to the total form in RAW264.7 macrophage cells; PB: purified bromelain, CB: crude bromelain. The results are expressed as the mean ± SD (*n* = 3). ^#^ *p* < 0.05 indicates a significant difference from the LPS-untreated cells, ** *p* < 0.005 and *** *p* < 0.001 indicate significant differences from the LPS alone.
